# Antioxidant and Antiviral Potential of Cold-Brewed and Cold-Concentrated Plant Extracts

**DOI:** 10.3390/ijms26199617

**Published:** 2025-10-02

**Authors:** Paulina Janicka, Damian Maksimowski, Aleksandra Chwirot, Maciej Oziembłowski, Katarzyna Michalczyk, Agnieszka Nawirska-Olszańska, Piotr Poręba, Sylwia Baluta, Ewa Kaczmar, Dominika Stygar, Barbara Bażanów

**Affiliations:** 1 Department of Pathology, Wrocław University of Environmental and Life Sciences, Norwida 31, 50-375 Wrocław, Poland; 2 Department of Functional Food Products Development, Wrocław University of Environmental and Life Sciences, Chełmońskiego 37, 51-630 Wrocław, Poland; damian.maksimowski@upwr.edu.pl (D.M.); maciej.oziemblowski@upwr.edu.pl (M.O.); 3 Department of Physiology, Faculty of Medical Sciences in Zabrze, Medical University of Silesia in Katowice, 19 H. Jordana Str., 41-808 Zabrze, Poland; katarzyna.michalczyk@sum.edu.pl; 4 Department of Fruit, Vegetable and Plant Nutraceutical Technology, Wrocław University of Environmental and Life Sciences, Chełmońskiego 37, 51-630 Wrocław, Poland; 5 Catalan Institute of Nanoscience and Nanotechnology (ICN2), CSIC and BIST, Campus UAB, Bellaterra, 08193 Barcelona, Spain; pporeba@icn2.net; 6 Institute of Advanced Materials, Wrocław University of Science and Technology, Wybrzeże Wyspińskiego 27, 50-370 Wrocław, Poland; 7 Department of Clinical Diagnostics, University of Warmia and Mazury in Olsztyn, Oczapowskiego Str. 2, 10-719 Olsztyn, Poland

**Keywords:** natural antioxidants, green extraction methods, antiviral, norovirus

## Abstract

Norovirus (NoV) is a symptomatic virus that is the leading cause of gastrointestinal disease. It spreads easily through the fecal–oral route and contact with contaminated food or surfaces. Maintaining a high level of hygiene in food industry settings and refocusing food production on isolating and testing natural compounds that exhibit antimicrobial and antioxidant properties are important elements in preventing NoVs infection. This study evaluated plant extracts prepared by cold brew and cold concentrate techniques for their antioxidant and antiviral activity. The extracts obtained demonstrated high antioxidant activity, with notable variation depending on the plant material, ranging from moderate to very strong levels. Correspondingly, high antiviral potential was observed, reaching the nearly complete inactivation of the virus. Remarkably, the highest virucidal effects were already achieved at relatively elevated, but not maximal, antioxidant activity levels. The results of the study indicate that cold water extraction techniques allow for the obtention of plant extracts showing strong virus-inactivating activity and favorable antioxidant activity.

## 1. Introduction

Plants contain significant amounts of bioactive compounds, particularly phenolic acids and flavonoids, which are drawing attention from the food industry for developing functional foods. Bioactive compounds also play an important role in preventing human disease. Plants’ bioactive compounds are water-soluble and most often heat-sensitive, so developing techniques with higher extraction efficiency is important. So far, many alternative extraction methods have been presented, such as microwave-assisted extraction, solvent extraction, ultrahigh-pressure extraction, ultrasound-assisted extraction, and organic and non-toxic solvent extraction. However water extraction is still considered the most beneficial [1], and thus cold water extraction techniques have gained enormous popularity worldwide and have become one of the main technological trends in the coffee and tea industry [2]. The results of a comparative analysis of the physicochemical properties of aqueous coffee extracts also appear to be relevant to the subject of this study. It was confirmed that coffee obtained using high-pressure carbon dioxide (HPCD) had the lowest CQA and caffeine recovery, whereas traditional extraction at an iced temperature was beneficial for the recovery of bioactive compounds—primarily polar ones, such as chlorogenic acids. It should be emphasized that the abundance of non-polar CO_2_ in the HPCD process reduced their partition coefficients from the raw material extraction matrix [3]. Thus, we investigated the use of this method to extract bioactive components. For example, polyphenols support the natural defense mechanisms of cells—the large number of hydroxyl groups present give these compounds antioxidant properties. In addition, due to their similar chemical structure to that of endogenous hormones, they have been shown to have a multidirectional effect on antivirals [4].

Foods and spices like oregano (*Origanum vulgare* L.), coffee (*Coffea arabica* L.), hemp (*Cannabis sativa* L.), thyme (*Thymus vulgaris* L.), nettle (*Urtica dioica* L.), rosemary (*Rosmarinus officinalis* L.), ginger (*Zingiber officinale* L. Roscoe), purple coneflower (*Echinacea purpurea* L.), cinnamon (*Cinnamomum verum* L.), and garlic (*Allium sativum* L.) are known to contain a wide spectrum of health-promoting components, like polyphenols and antioxidants [5]. These compounds regulate reactive oxygen species (ROS) levels and impact the antioxidant system of the body [6]. They are also known for their anti-inflammatory and antiviral properties [7]. Numerous viruses, including the measles virus, hepatitis C virus, herpes simplex virus, and influenza virus, have been examined in relation to polyphenols’ antiviral activity [8,9,10]. In addition, the antibiotic and antiparasitic properties of raw materials and their aqueous extracts have also been widely described in the literature [11]. Oregano has been utilized for wounds, headaches, depression, digestive and respiratory ailments, as well as stomach and expectorant issues [12]. Coffee, apart from its popular consumption, is widespread all over the world because of its pharmacological role. Numerous studies indicate that low caffeine-to-chlorogenic acid ratios may reflect enhanced health-promoting properties and protect against the development of civilization diseases. [13,14]. Hemp has been used to treat oral and dental diseases in China since 2700 BC [15]. It has been used as an analgesic in the treatment of tetany and convulsions, gout, malaria, insomnia, shortness of breath, and cough. It has also been used for headaches, irregular menstruation, pruritus, and anemia [16]. Thyme has a long history in the treatment of respiratory diseases. It has been used for whooping cough, bronchitis, and asthma [17]. In folk medicine, it has also been used for the mouth, stomach, intestines, and respiratory tract infections and to strengthen the heart [18]. Nettle is considered a diuretic and an astringent and is also used to treat coughs, colds, asthma, and jaundice, whereas the stem’s juice is used for fever [19]. The plant can also be applied topically on cuts, burns, wounds, and to treat boils and blisters [20]. Rosemary is administered to patients to treat headaches, abdominal pain, painful menstruation, epilepsy, rheumatic pain, and spasms, to improve memory, and to alleviate nervous agitation, attacks of hysteria, or depression [21,22]. Ginger plays an important role in traditional Ayurvedic medicine [23]. It has been used especially for cough and asthma but also for indigestion, loss of taste and appetite, flatulence, nausea, vomiting, allergic reactions, acute and chronic coughs, cold, fever, allergic rhinitis, sinusitis, acute or chronic bronchitis, respiratory problems, headache, back muscle pain, toothache, and swollen gums [23]. Purple coneflower has been used to treat various infections and wounds and has become a very popular herbal medicine for humans and animals around the world [24]. In addition, coneflower may prove to be a better natural medicine ingredient than garlic due to its better olfactory properties, which are more desirable to consumers [25,26]. Cinnamon has long been used as an antiemetic, antidiarrheal, and antiflatulent stimulant [27]. In addition, it is used in aromatherapy to improve mood [28]. Garlic bulb is used worldwide for hypertension, colds, malaria, cough, pulmonary tuberculosis, sexually transmitted diseases, mental disorders, kidney and liver diseases, asthma, and diabetes, and as a snakebite remedy [29]. In addition to lowering blood pressure, cholesterol, and glucose levels, it also prevents blood clotting [30]. Moreover, Singh et al. described its anticancer activity [31]. The literature reports strong the virus-reducing effect of garlic against Coxsackie, herpes simplex types 1 and 2, influenza B, parainfluenza type 3, cowpox, vesicular stomatitis, HIV-1 and 2, and human rhinovirus type 2 [29], which, in the context of limited antiviral treatment options, seems particularly valuable.

One of the most common causes of viral gastroenteritis is noroviruses (NoVs) [32,33], for which no approved antiviral drugs or licensed vaccines are available and only symptomatic treatment is used [34]. NoVs are non-enveloped, positive-sense, single-stranded RNA viruses (+ ssRNA viruses) belonging to the *Caliciviridae* family. It is estimated that they are responsible for 64,000 hospitalizations per year [35].

Despite so many hospitalization cases and deaths, the most common symptoms of NoV infection include diarrhea, vomiting, abdominal pain, fever, and headache [36]. In some cases, neck stiffness, stupor, and photophobia appear [32]. The average duration of symptoms is 2–3 days from the onset of the disease. Nowadays, patients are increasingly turning to natural compounds, which can significantly shorten the duration of diseases or even prevent them [37], particularly to fight against the most common causes of viral gastroenteritis [32,33].

For example, a study by Hayashi et al. [38] showed that monogalactosyl diacylglycerol from the green microalga *Coccomyxa* sp. showed antiviral potential against murine norovirus and feline calicivirus (FCV), indicating that some microalgal compounds can effectively inactivate norovirus. In addition, phenolic compounds (particularly quercetin), together with essential oils from allspice and lemongrass, showed significant virus-reducing effects in vitro against MNV, suggesting that these natural products may serve as potential antiviral agents [38,39]. Studies by Lim et al. [40] further confirm that plant extracts can inhibit norovirus activity under simulated digestive conditions [40,41]. This is consistent with the work of Iloghalu et al. [42], who reported that extracts from *Hibiscus sabdariffa* also showed antiviral activity against MNV, reflecting the potential of various plant extracts in controlling norovirus infections. Furthermore, the antiviral efficacy of seaweed extracts was investigated, and Kim et al. [43] found that methanolic extracts from *Undaria pinnatifida* showed significant virus-inactivating activity against FCV, which serves as a norovirus surrogate. Similarly, Eom et al. [44] highlighted the antiviral activity of dieckol and florofucofuroeckol-A from the brown alga *Eisenia bicyclis* against MNV, further underscoring the role of marine plants in norovirus inactivation. Gilling et al. [39] noted that while lemongrass oil and citral can reduce viral infectivity, they do so through different mechanisms, suggesting that the efficacy of plant oils may depend on their specific chemical properties.

The mechanisms by which plant-derived compounds exert their antiviral effects are diverse. This study aimed to develop novel cold extraction techniques against different types of plant raw materials, and determine their virus-neutralizing effect and antioxidant activity. The study assumed that a controlled cryoconcentration process can improve the antiviral properties of extracts obtained by the cold brew technique by promoting the activity of antioxidant compounds.

## 2. Results

In the food industry, the extractability of nutrients and their sensory characteristics are essential. In our study, we focused on the amount of dissolved solids in the aqueous solution depending on the ten plant materials, which may also have practical relevance for process yield calculations and is the starting point for the isolation and analysis of bioactives from plant materials [45].

Generally, the TDSs increased after the concentration process for all cold brew samples (Table 1) and ranged from 0.77% to 1.01% TDS. The differences with the highest concentration value were observed for oregano leaves (2.02) and the lowest for cinnamon bark (1.49). Among the results, thyme leaves showed high process yields with a value of 1.89. These comprise one of the matrices that provide a wide range of biomolecules, such as phenolic compounds and sucrose, which are very stable in different conditions [46].

The phenolic compounds present in our raw materials were mainly phenolic acids and flavonoids [47]. One of the most popular methods for determining these compounds in plant extracts is the fast and efficient spectrophotometric method [48]. The TPC varies in all raw materials due to the wide concentration range, with concentrations depending on particular plant material. In a similar research topic, with slightly different materials, the TPC range was 48.86 mg GAE/100 mL for lemon balm and it was much higher than in sage (27.94 mg GAE/100 mL) [49]. In our study, the TPC ranged from 105.73 mg GAE/100 mL (for oregano extract) to 9.11 mg GAE/100 mL (for garlic extract) (Table 2). Ginger and rosemary extracts presented relatively similar TPC values of 24.13 and 24.83 mg GAE/100 mL, respectively. The same was observed for garlic and cinnamon extracts with TPC values of 9.11 and 9.63 mg GAE/100 mL, respectively. The TPC measured in hemp (seeds and leaves) of 47.26 mg GAE/100 mL exceeded the TPC of all other samples and was lower only compared to that of oregano extract (105.73 mg GAE/100 mL) and coffee extract, for which the TPC value was the second highest among the results (77.67 mg GAE/100 mL).

In our study, oregano and coffee extracts presented higher FC concentrations, of 1217.94 mg/100 mL and 955.28 mg/100 mL, respectively (Table 2), whereas the other plant extracts contained four times less flavonoids, compared to oregano, and three times less flavonoids compared to coffee extract.

Regarding antioxidant capacity, roasted coffee presented the lowest antioxidant capacity. The average value for other herbs and spices was 785.84 μmol Trolox/100 mL. Only oregano and thyme leaf extract showed an antioxidant capacity below the average for other herbs and spices extracts, of 574.56 μmol Trolox/100 mL and 762.45 μmol Trolox/100 mL, respectively. The highest antioxidant capacity was found for the garlic bulb sample: 844.72 μmol Trolox/100 mL.

The highest values (99.99%) for antiviral potential were achieved by purple coneflower, garlic, cinnamon, ginger, thyme, rosemary, and nettle, followed by hemp seed (99.95%), coffee (99.91%) and oregano (99.90%) extracts. For EPI and CQA, the reference substances, the virus-neutralizing effects were 0% and 99.73%, respectively. Regarding cytotoxicity, the plant extracts tested showed no CPE on the RAW 264.7 cell line at a dilution of 10^−3^ (Table 3), while EPI and CQA exhibited no toxicity at a dilution of 10^−2^. Therefore, these dilutions (concentrations of 18% and 10%, respectively) were used in our antiviral studies.

**Figure 1:**
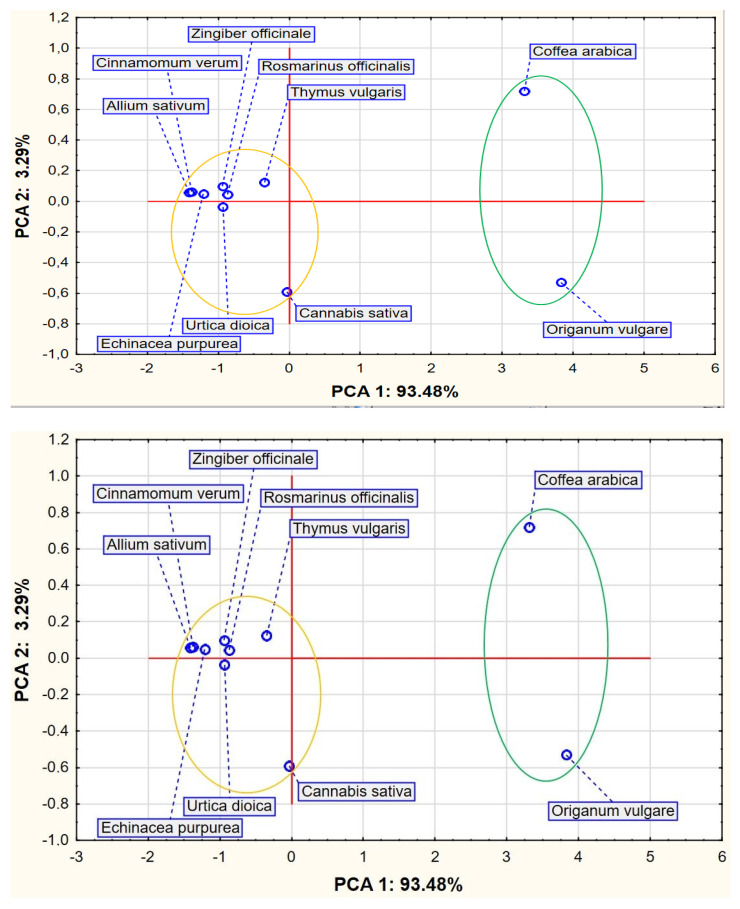
Chart of factor coordinates of cases (plant extract source) in the PCA model.

The obtained results allowed us to performed our analyses. The input data referred to ten cases (plant extract source) and four variables as average values from Table 2. PCA showed that the first two PCA factors (i.e., PCA1 and PCA2) explained 96.77% of the total variance. The eigenvalues were 3.29% and 93.48% for PCA1 and PCA2, respectively. An analysis of Figure 1 indicates the existence of two compact clusters of points representing the analyzed variants. The first group consists of the ‘*Coffea arabica*’ and ‘*Origanum vulgare*’ plant extract source, and the second group consists of the rest of the variants.

**Figure 2:**
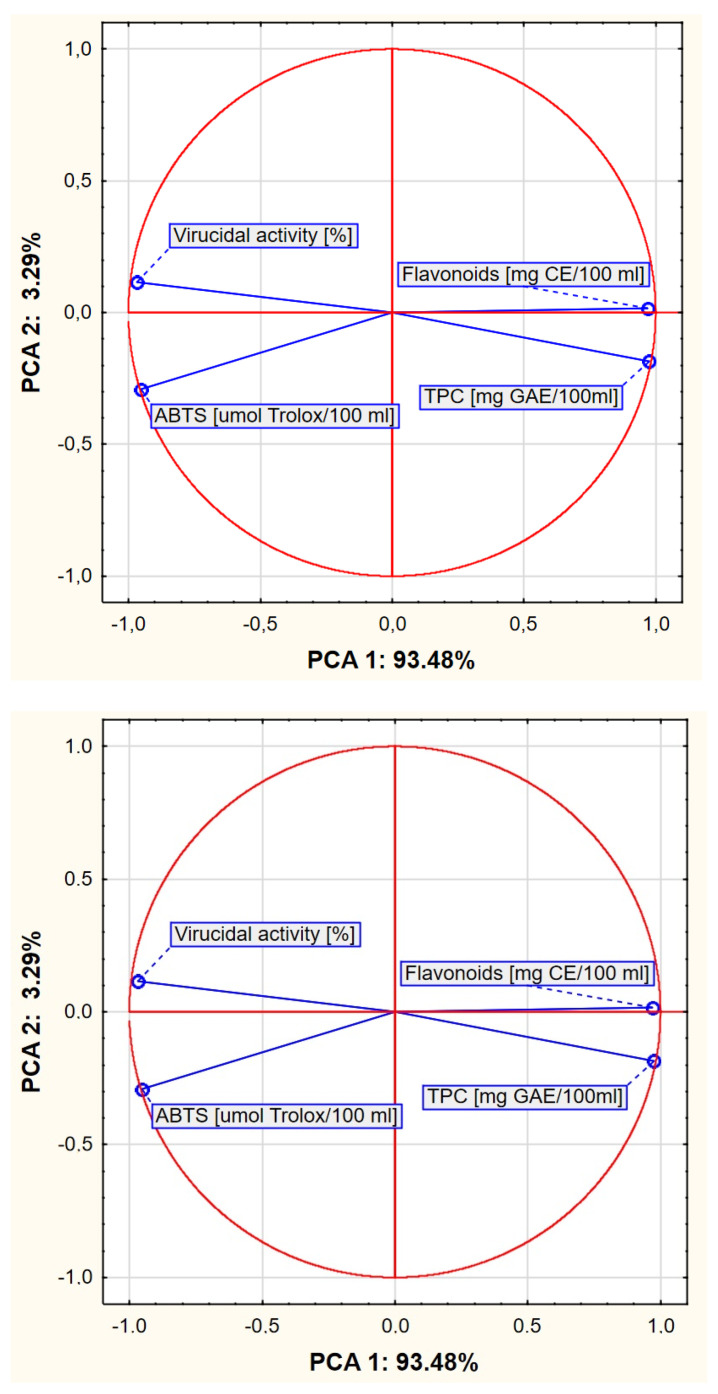
Chart of factor coordinates of variables in the PCA model.

When interpreting factor coordinate plots of variables (Figure 2), the length of the vectors and the angle between them are considered. The longer the vector, the greater the contribution of the trait (or variable, here TPC, flavonoids, ABTS, and antiviral potential) to the values of the two components analyzed (here PCA1 and PCA2). Also, a similar effect of these traits (variables) occurs when there is a smaller angle between the vectors. This analysis facilitates the prediction of correlations between the antioxidant and antiviral properties of cold-concentrated plant extracts, which may be important for further research. According to the experiment, a stronger positive correlation was observed between TPC and flavonoid concentration compared to the relationship between the virus-reducing effect and antioxidant activity. An angle close to 180° between TPC and virus-inactivating activity indicates that variables are negatively correlated.

Although polyphenolic compounds and flavonoids are well recognized for both their antioxidant potential and their ability to modulate viral infectivity, it is important to emphasize that these two types of activity are not synonymous. Antioxidant activity reflects the ability of compounds to scavenge free radicals or modulate redox balance, whereas virus-inactivating effects involve direct or indirect interference with the viral particle or replication cycle. Our findings, in which extracts with very different TPC and flavonoid contents all achieved > 99.9% virus inactivation, indicate that the antiviral effect cannot be attributed solely to antioxidant capacity. This suggests that additional bioactive molecules, synergistic interactions, or distinct mechanisms unrelated to radical scavenging are likely involved, which should be addressed in future mechanistic studies.

## 3. Discussion

It can be assumed that supplementing extracts made from raw plants helps alleviate the symptoms or shorten the duration of diseases. Secondary metabolites (i.e., phenolic compounds, flavonoids, alkaloids, terpenes) are known for their significant biological properties and potential activity against various human viruses. They also present antioxidant and anti-inflammatory properties [50].

A significant TDS ratio was observed for coffee. This can be explained by the earlier processing—intense heat during roasting green beans damages the cellular matrix, making the soluble compounds easier to extract in cold water [51]. Contrarily, the recovery of the TDS% after the concentration process may have decreased due to the occlusion of bioactive compounds [52], as well as the position of functional groups, resulting in variations in chemical properties, which can influence the solubility and freezing of these compounds [53].

Flavonoids are important natural bioactive compounds in preventing and treating diseases. They are also pigments that give plants different colors, from yellow in citrus fruits to dark blue in berries. The consumption of fresh fruit and vegetables in our diets is the main source of these nutrients; the average daily intake is 1 g. They are also present in stimulants (tea, red wine) [54]. For example, Hosu et al. [55] classified wines (variety/vineyard/year) in terms of flavonoid content. The authors determined FC levels ranging from 0.67 to 29.10 mg/100 mL in the first year after production and from 1.03 to 29.12 mg/100 mL in the third year after production. Rana et al. [56] found that the concentration of flavonoids ranged from 91 to 200 mg/100 g for apple pomace dried with different methods. Differences in the flavonoid content were also observed in different parts of the apple: concentrations in the skin reached 47.8 mg in 100 g of fresh material, and in the pulp, it was 16.0 mg of quercetin equivalents [57].

In the present study, we observed a negatively correlated relationship between TPC and antioxidant activity. The reason for this phenomenon may be the relatively high content of sugars or melanoid components in coffee, which may result in this phenomenon occurring disproportionately, with a negative impact on the ABTS cation radical scavenging capacity [58]. This effect may also result from the oxygen degradation of some ingredients due to the high surface contact of the cryo-concentrate with air during the gravitational collection of the product from the ice block. On the other hand, the Folin–Ciocalteu reagent measures the total reducing capacity of the sample, so not all components dissolved in the extract characterize the antioxidant activity [59]. Also, Ziarno et al. [60] observed no correlation between the TPC and the antioxidant activity of selected spices and their aqueous extracts. Skotti et al. [61] tested aqueous extracts from selected Greek plants. For example, the following results were obtained for oregano extract at various temperatures: 85 °C (TPC 0.64 mg/mL; antioxidant activity 3.35 µmol Trolox/mL); room temperature (TPC 0.32 mg/mL; antioxidant activity 1.56 µmol Trolox/mL); and room temperature with the assistance of ultrasounds (TPC 0.34 mg/mL; antioxidant activity 1.73 µmol Trolox/mL). Our results indicate a slightly higher ABTS radical scavenging capacity of the aqueous extracts (antioxidant activity 5.74 µmol Trolox/mL). It’s worth noting that prolonged extraction times during cold brewing under controlled conditions can enhance the extraction efficiency of antioxidant compounds from plants. Asensio et al. [12] evaluated three species of oregano—*O. majoricum*, *O. vulgare* ssp. *vulgaris* and *O. hirtum*. Antioxidant values of the essential oil ranged from 26.569 to 54.82 µmol Trolox/100 mL and from 0.234 to 0.163 mMTrolox/mg. We obtained 574.56 µmol Trolox/100 mL in our analysis, which is more than ten times that found in Asensio et al.’s work [12]. This could be because they looked at essential oils rather than plant extracts. Generally, the antioxidant capacity can be explained by the amounts of phenolic compounds found in the sample. However, results obtained for water extracts measured by spectroscopy (ABTS+, FRAP, DPPH) can be overestimated in samples containing a low concentration of phenolic compounds with a high content of ascorbic acid, which is an interfering substance [62]. For example, rutin and ascorbic acid are detected in teas and herb plants at 2.8% and 3.5% concentrations, respectively [63].

In the case of coffee, many components during the roasting of green beans have been attributed to the thermal degradation of some phytochemicals [64], whereas antioxidative effects can boost the presence of melanoidin compounds depending on roasting parameters (higher temperature increases their concentration up to a certain point) [65]. In a different study, the antioxidant capacity of cold-brewed coffee extracts ranged from 1336 to 1745 µmol Trolox/100 mL [66]. These results were higher than those in the present study (451.15 µmol Trolox/100 mL) due to the use of a five-fold higher dose of coffee for extraction. Sukoco et al. [65] compared the antioxidant capacities of caffeinated and decaffeinated coffee and ginger. The baseline antioxidant activity of caffeinated coffee was 40 mmol/100 mL, and that for decaffeinated coffee was 17 mmol/100 mL. An addition of 10% ginger increased the values by 2 and 1 mmol/100 mL. Their values were extremely high, and this may be due to different coffee species, as well as the roasting and extraction conditions. Nevertheless, in our study, coffee extract presented the second highest TPC and flavonoid content values. Hence, further analysis is needed to prove that there are increased antioxidant properties as well as increased antiviral properties. However, the antiviral potential of coffee extract obtained by the cold brew technique was 99.91%. We regard this as a reflection of the highly inhibitory effect on norovirus, contrasting similar applications of this technique in other research, where the coffee extracts in ethanol solutions were not effective for any of the viruses studies [67]. Results from the study by Utsunomiya et al. [68] suggest that caffeic and chlorogenic acids have the best antiviral properties and can inhibit both DNA and RNA viruses, while the potential for the pharmacological use of roasted coffee depends on the method used to extract the bioactive constituents.

Smeriglio et al. [69], studying the same variety of hemp as we did, noted the antioxidant capacity of this plant at 695 μmol Trolox/100 mL, which is less than 100 μmol/100 mL lower than that obtained in our study. On the other hand, the antibacterial properties of leaf extracts of different varieties of *Cannabis sativa* L. have already been observed to be at levels lower than 100 μmol/100 mL [70], so the above difference can be considered insignificant. Analyzing the results of our study, it is worth noting that the sample was concentrated only twice—if the concentration level increased, better analytical results could have been achieved.

Our results are in agreement with those obtained by Abdelfadel et al. [71], who reported similar concentrations of TPC compounds in thyme. Thyme was also studied by Köksal et al. [72], who used different organic solvents for antioxidant extraction. The researchers reported that the antioxidant capacity of an aqueous extract of thyme was 89 μmol/100 mL, whereas ethanol extract showed a significantly higher value of 1602 μmol/100 mL. Although cold brewing is a method that uses a low temperature and that only requires a long extraction time, it can allow for the efficient extraction of bioactive compounds (762.45 μmol/100 mL), as highlighted by the comparison of aqueous extracts mentioned above. Taking this into account, optimization of the cold brew process can increase the extraction efficiency of soluble components, achieving results comparable to those obtained with organic solvents.

Different aqueous and alcoholic extracts of nettle have been studied [73]. Leaf extract brewed with boiling water and via infusion prepared at 60 °C for 3 h showed high antioxidant values, 210 and 268 μmol, respectively, while in leaf extract obtained in 70% ethanol, these values were higher, at 310 μmol. In our study, mixed plant parts were used for extraction, which may have led to different properties reflecting antioxidant activity. On the other hand, convection drying using hot air may lead to changes in the tested activity, caused by high drying intensities and the associated loss of bioactive compounds. However, considering the antioxidant capacity of nettle in our research, high-bioactivity ABTS+ was achieved at 818.32 μmol Trolox/100 mL, as was a virus-neutralizing effect at 99.99%.

The antioxidant activities described in the literature for a single rosemary extract measured according to three methodologies, ABTS, DPPH, and FRAP, were 38, 513, and 662 μmol Trolox/100 g, respectively [74]. In another study, Soxhlet extraction, maceration, and ultrasonication yielded antioxidant capacities of 1270, 910, and 810 μmol/100 mL for rosemary extract, respectively [75]. In our analyses, rosemary extract presented an antioxidant activity of 807.27 μmol/100 mL. It is reasonable to assume that rosemary extract obtained by cold extraction has a similar bioactive antioxidant capacity to that mentioned above for rosemary extract obtained via maceration and ultrasonic extraction. Thus, if rosemary is considered a virus-inactivating activity unit, optimizing the costs of technological application can be considered a factor in scaling production because, for three different methods, the results seem to be similar.

Sida et al. [76] studied the physicochemical properties of ginger. They found that the analyzed material presented high antioxidant capacity (42.23–223.50 μmol Trolox/g) and low TPC as well (5.08–12.21 μmol Tannic acid/g). In our study, we obtained very good parameters, both in terms of antioxidant (798.67 μmol/100 mL) and virus-reducing (99.99%) properties. Thus, we can conclude that dried ginger root or ginger extract can enhance the antioxidant capacity and production efficiency of medications or supplements, which is very beneficial to health.

The performed research also highlighted the potential use of purple coneflower extracts as a source of valuable compounds with antioxidant properties. The combined effect of the variables (extraction temperature and concentration process temperature) indicates high antioxidant activity of 832.44 μmol Trolox/100 mL, with an extremely low TPC of 14.54 mg GAE/100 mL. Therefore, this study may constitute a reference point for the extraction process under thermal conditions and the potential application of this raw material as a high-performance and safe component as a source of phenolic compounds after condition optimization.

Dvorackova et al. [77] analyzed cinnamon antioxidant activity obtained by ultrasound extraction (260 μmol/mL). Significant differences between ultrasound-assisted extraction and conventional cold brew methods were found in the chemical and physical parameters and sensory profiles [78], whereas our virological studies indicate that cold concentration, esspecialy made from cinnamon extract is sufficient to achieve a complete virus-reducing effect. However, comparable TPC levels were found for cinnamon by Wijewardhana et al. [79], with a difference of 6.59 mg GAE/100 mL.

The chemical complexity of garlic extract meant that it had the lowest TPC (9.11 mg GAE/100 mL) and flavonoid content (259.08 mg CE/100 mL) compared to the other substances tested, but paradoxically, it had the highest antioxidant capacity (844.72 μmol/100 mL) and 99.99% virus-inactivating action. Based on the results, garlic was found to be the most effective extract of bioactive compounds with antioxidant properties from the applied technique processed under non-thermal conditions.

Recent studies on the very popular components of elderberry tea (*Sambucus nigra* L.) proved that it exerts antiviral bioactivity against the vaccinia virus, reducing its infectious titer by up to 95% [80]. Pachura et al. [81] analyzed the leaves of *Ilex paraguariensis* (known as Yerba mate, used as a popular beverage) and investigated its underlying antiviral and cytotoxic properties. The study showed significant activity against HSV-1 and HAdV-5 viruses, achieving a 99.99% reduction in infection. The virus-neutralizing effect of all our tested natural plant extracts was also over 99.9% (Table 2).

Polyphenols may strengthen the body’s defenses against norovirus infections indirectly by influencing intestinal permeability and microbiota. Moreover, polyphenols have been discovered to possess antioxidant and anti-inflammatory qualities, which may help the immune system against viral infections [82]. Norovirus, a huge global public health burden, is very hard to prevent and control because of its low infectious dose and stable habitat [83]. According to Tsai et al. [84], polyphenols specifically work against noroviruses by disrupting protein synthesis and triggering host cell antiviral response pathways. The JAK/STAT signaling pathway and RNA polymerase II-mediated transcriptional responses that inhibit norovirus replication in human intestinal epithelial cells may be modulated by polyphenols to produce their antiviral effects [85].

The type of virus and its structure determine the mechanism of action of polyphenols in virus control [86]. In the case of herpes simplex virus type 1 (HSV-1), polyphenols block host cell glycoproteins during the attachment and fusion steps and the virus’ spread between body cells [87]. Diaz et al. [88] showed that the levels of phenolic and flavonoid compounds of medicinal plants were correlated with their anti-inflammatory and antioxidant effects. This is probably why phenolic phytochemicals have long been used by humans to treat a wide range of ailments, including bacterial, protozoan, fungal, and viral infections, inflammation, diabetes, and cancer [89]. For example, the flavonoid scutellarin from the root of *Scutellaria baicalensis* L. has been shown to inhibit the nsP13 helicase of the SARS-CoV-2 virus by altering its ATPase activity [90]. According to Hendrayana studies, polyphenols can obstruct the virus’s ability to replicate in a number of ways, including blocking the virus’s ability to replicate and transcribe, enter host cells, and mature [91].

Vazquez-Calvo et al. [92] used polyphenols found in green tea and wine to analyze their effects on West Nile virus (WNV), Zika virus (ZIKV), and dengue virus (DENV). They found that the polyphenols delphinidin and epigallocatechin gallate showed the greatest antiviral properties against WNV at a concentration of 10 μM. To determine the effect on the virus, both polyphenols were added at different times of infection. Both compounds were found to affect the early stages of infection. The LysoSensor assay confirmed the direct virus-reducing effect of the above-mentioned substances on the virus particle. The described compounds also showed virucidal activity against ZIKV and DENV [92].

According to mentioned research by Bahramsoltani et al. [9], polyphenols offer a wide range of antiviral properties because they have been demonstrated to prevent viral adherence, replication, hemagglutination, and penetration into host cells. Furthermore, these substances have the ability to alter cellular signaling pathways, including the protein kinase C pathway, in order to carry out their antiviral actions [10]. Moreover, polyphenols’ ability to promote both general immune function and defense mechanisms against viruses is highlighted by their impact on gut health. These compounds can affect intestinal permeability as well as prevent diseases like obesity and dysbiosis by regulating oxidative and inflammatory processes dependent on gut microbiota [93].

Although there are a number of papers on the antiviral potential of oregano, thyme, nettle, rosemary, ginger, and cinnamon [39,94,95,96,97] against noroviruses, all of them, like ours, involve in vitro studies, which may not be directly applicable to the human or animal body. Disregarding technical differences regarding the form of the extracted substance, all these studies yielded reductions between 1.07 log (oregano) and 4 log (nettle) [98]. These results are in line with ours, in which oregano extract also showed the lowest virus reduction, while nettle extract was one of the most effective in terms of antiviral potential.

## 4. Materials and Methods

### 4.1. Plant Extracts

The plant material (oregano leaves, hemp seeds and leaves, thyme leaves, nettle leaves, rosemary leaves, ginger root, purple coneflower leaves and flowers, cinnamon bark, garlic bulb) used for the experiment was purchased at commercial maturity from the herbal shop (Hala Targowa, Wrocław, Poland). Roasted coffee beans were obtained from the local Coffee Roastery in Wrocław (Etno Cafe, Wrocław, Poland).

Plant extracts were prepared using a two-stage process: the first stage involved cold brewing [99], and was followed by cryoconcentration.

#### 4.1.1. Cold Brewing Technique

One gram of plant material was mixed with 49 mL of RO (reverse osmosis) water and infused for 12 h at 20 °C (±1 °C). Then, each infusion was filtered using a Hario coffee paper filter V60, 01 Size, 100 pcs (Hario, Koga, Japan).

#### 4.1.2. Cryoconcentration (Cold-Concentration)

Cryoconcentration was performed using the Maksimowski method [100], with slight modifications. The filtrates were frozen for one day at −18 °C in 50 mL conical tubes in a Labcold freezer-type RLVF1517 (Labcold, Basingstoke, UK). Then, the samples were placed in a SAMSUNG RB34T671FSA EF No-Frost refrigerator (SEPM, Wronki, Poland), where they were placed upside down on a stand. At a temperature of 5 °C, the samples were thawed within 5 h until half of the volume was collected before freezing. They were frozen again to −18 °C until the analytical tests were performed.

#### 4.1.3. Total Dissolved Solids (TDSs)

TDS assays in all cold-brewed and cold-concentrated plant extracts were measured using a digital refractometer (Atago, Tokyo, Japan). When the temperature stabilized, the instrument analyzed each sample and the results from the refractometer were represented as Brix (%) and calculated automatically using the following equation [101]:

Total Dissolved Solids (%) = Brix (%) × 0.85

The analysis was repeated in triplicate, and the results were shown as an average of the amount of total dissolved solids.

#### 4.1.4. Total Phenolic Content (TPC)

Total phenolic content was determined using a UV spectrophotometer 2401 PC (Shimadzu, Kyoto, Japan) via the Folin–Ciocalteu (FC) method and expressed as milligrams of gallic acid (GAE) per 100 mL [37]. The cuvettes containing 0.10 mL of the freshly mixed individual plant extract sample, 0.20 mL of FC reagent, 2.00 mL of distilled water, and 1.00 mL of Na_2_CO_3_ (10%) were left in the dark for 1 h before analysis. All concentrated samples were measured spectrophotometrically in triplicates against distilled water absorbance at λ = 765 nm.

#### 4.1.5. Flavonoid Content

Flavonoid content was determined using a UV spectrophotometer 2401 PC (Shimadzu, Kyoto, Japan) in accordance with Diaz et al. [88], with respective modifications. The plant extract (0.50 mL) was added to a 10.00 mL test tube containing 2.00 mL of distilled water and mixed with 0.15 mL of 5% NaNO_2_. After 5 min of incubation, 0.15 mL of 10% AlCl_3_ 1.00 mL was added, and after 3 min, the reaction mixture was treated with 1 mL of 1 M NaOH. Finally, the volume was adjusted to 5.00 mL with distilled water, and the mixture was incubated for 10 min at room temperature. Finally, the absorbance was measured at λ = 510 nm against a blank control sample, and the results were expressed as mg of catechin equivalent (CE)/100 mL. All concentrated samples were analyzed in triplicates.

#### 4.1.6. Antioxidant Activity

Antioxidant activity was determined using a UV spectrophotometer 2401 PC (Shimadzu, Kyoto, Japan) by observing the decrease in ABTS+ cationic radical levels and expressed as μmol Trolox per 100 mL. Samples measuring 5.00 mL of plant extract were centrifuged for 5 min at 7000 rpm. Then, 0.03 mL of the supernatant was transferred to a spectrophotometric vial, mixed with 3 mL of ABTS solution and incubated at room temperature for 6 min. A decrease in the absorbance was measured at λ = 734 nm against a blank control sample [102]. All concentrated samples were analyzed in triplicates.

### 4.2. Virological Tests

#### 4.2.1. Cell Culture for Virological Test

In vitro experiments were performed using RAW 264.7 macrophage cells (TIB-7^TM^, ATCC, Manassas, VA, USA) cultured in Dulbecco’s Modified Eagle’s Medium (DMEM) with nonessential amino acids, with the addition of 10% fetal bovine serum (FBS) (Biological Industries, Kibbutz Beit-Haemek, Israel). The cells were incubated in 25 mL polystyrene flasks (Googlab Professional Line, Rokocin, Poland) at 37 °C with 5% CO_2_ and at 95% humidity (CellXpert^®^ C170i, Hamburg, Germany) and passaged using 0.25% trypsin-EDTA (Biological Industries, Kibbutz Beit-Haemek, Israel).

#### 4.2.2. Virus Propagation

Murine norovirus (MNV) (VR-1973, ATCC, Manassas, VA, USA) was used in this study. When the RAW 264.7 cells reached 75–80% confluence, the medium was removed, and the cells were washed with PBS. Fifty microliters of MNV suspension TCID_50_ (Tissue Culture Infectious Dose 50%) was added to the flask and incubated for 3 h at 37 °C with 5% CO_2_. After that, the virus suspension was removed, the cell culture was washed with PBS, and DMEM was added. Cells were incubated for 4 to 6 days and observed daily using an inverted microscope (Axio Observer, Carl Zeiss MicroImaging GmbH, Jena, Germany) to detect the cytopathic effect (CPE).

Infectivity was determined by the endpoint titration, in which 0.1 mL of each dilution was transferred into 8 wells of a microtiter plate, starting with the highest dilution. This was followed by the addition of 0.1 of freshly trypsinized RAW 264.7 cells (2 × 10^5^ cells/mL). Microtiter plates were incubated at 37 °C in the 5% CO_2_ atmosphere. The plate was observed every day (up to 5 days) and the cytopathic effect was read by using an inverted microscope. Calculation of the infective dose TCID_50_/mL was performed with the Spearman and Karber method using the following formula:

−log_10_TCID_50_ = x_0_ − 0.5 + Σ r/n

where TCID_50_—tissue culture-infectious dose; x_0_—log_10_ of the lowest dilution with a 100% positive reaction; r—number of positive determinations of the lowest dilution step with a 100% positive reaction and all higher positive dilution steps; n—number of determinations for each dilution step.

### 4.3. Cytotoxicity Assay

RAW 264.7 cells with 70% confluence were seeded into 96-well plates. Serial dilutions from 10^−1^ to 10^−8^ of all plant extracts tested and reference substances—epicatechin (EPI) and caffeoylquinic acid (CQA)—were applied to the cell culture in 3 replicates. The plates were incubated for 2 days in 37 °C, 5% CO_2_. After this time, the cytotoxic effect of the plant extracts was observed. Three technical repetitions were performed for each compound.

### 4.4. Antiviral Potential

The virus-inactivating activity of the plant extracts was tested using European Standard EN 14476 [103]. Briefly, 1 volume part of the test virus (MNV) suspension (0.1 mL of 1 × 10^−8^ TCID_50_), 1 volume part of the interfering substance (0.1 mL of PBS), and 8 volume parts of the tested substance in a concentration that was non-toxic to RAW 264.7 cell culture (Table 4) were mixed, and then after 1 h under room temperature, aliquots were taken and serial dilutions up to 10^−8^ were prepared for each mixture. Subsequently, 50 µL was added (virus and extract) from each dilution in eight replicates to wells of a 96-well plate containing a confluent monolayer of RAW 264.7 cells. The plates were observed using the inverted microscope daily for up to 4 days for the development of viral cytopathic effects. In parallel, the titer of the control virus was determined, and additionally, according to the same procedure, EPI and CQA reference substances were tested as controls. Then, the residual infectivity was calculated. According to PN-EN 14476, a substance has virucidal properties if, within the recommended exposure time, the titer is reduced by ≥4 log_10_ steps (inactivation ≥ 99.99%) compared to the titer of the control virus.

### 4.5. Statistics

The obtained data were subjected to statistical analysis using Statistica 13.0 (StatSoft Polska, Kraków, Poland). They were recorded as means ± standard deviation (SD). One-way analysis of variance was conducted where the means in the post hoc test were compared using Duncan’s test (statistically significant differences were those at *p* < 0.05). Principal component analysis (PCA) and a correlation between the selected properties of the samples were also performed.

## 5. Conclusions

Extracts of commonly available food plants show virus-reducing effect properties against noroviruses and may, therefore, be useful in supplemental treatment and the reduction in infection with this virus.

All tested natural plant extracts showed virus-inactivating activity above 99.90%. The highest values (99.99%) presented the extracts from purple echinacea, garlic, cinnamon, ginger, thyme, rosemary and nettle, followed by extracts from hemp seeds (99.95%), coffee (99.91%), and oregano (99.90%).

The PCA indicated the similarity of the analyzed properties between *Coffea arabica* L. and *Origanum vulgare* L. plant extract sources, which constitute one group, as well as among the rest of the plants, which constitute the second group.

The cold brew and cold concentrate methods allow for the obtention of plant extracts with high antioxidant activity, which is very important when using these extracts to enrich food with active ingredients.

Due to their high virus-neutralizing effects and, at the same time, the fact that they contain the best antioxidant properties among the plants tested, cryoconcentrates of garlic, Ceylon cinnamon and purple echinacea extract seem to be the most suitable for use as ingredient-enriching foods, with active ingredients counteracting NoV infection.

Nevertheless, further research is needed into their mechanisms of action, and their safety and efficacy in vivo. In particular, it will be important to develop appropriate doses and application forms of these extracts to ensure their clinical efficacy. In addition, future studies should include a wide range of other viruses and interactions with conventional antiviral therapies to determine their full therapeutic potential.

## Figures and Tables

**Table 1:** Total dissolved solids [%] of the cold brewed and cold concentrated plant extracts.

Plant Extract Source	TDS Extract	TDS After Concentration	Ratio TDS (After Concentration:Extract)
	[%]		
*Origanum vulgare* L. Oregano leaves	0.48 ± 0.07 ^d^	0.97 ± 0.12 ^b,c^	2.02
*Coffea arabica* L. Arabica coffee roasted beans	0.53 ± 0.04 ^c^	0.99 ± 0.06 ^a^	1.86
*Cannabis sativa* L. Hemp seeds and leaves	0.56 ±0.02 ^b^	1.01 ± 0.06 ^a^	1.80
*Thymus vulgaris* L. Common thyme leaves	0.49 ± 0.08 ^d^	0.93 ± 0.02 ^c^	1.89
*Urtica dioica* L. Common nettle leaves	0.41 ± 0.05 ^e^	0.77 ± 0.07 ^e^	1.87
*Rosmarinus officinalis* L. Rosemary leaves	0.52 ± 0.04 ^c^	0.94 ± 0.01 ^c^	1.81
*Zingiber officinale* Roscoe Ginger root	0.49 ± 0.08 ^d^	0.81 ± 0.04 ^d^	1.65
*Echinacea purpurea* L. Purple coneflower	0.54 ± 0.05 ^b,c^	0.96 ± 0.02 ^b,c^	1.77
*Cinnamomum verum* J. Presl Ceylon cinnamon bark	0.63 ± 0.03 ^a^	0.94 ± 0.10 ^c^	1.49
*Allium sativum* L. Garlic bulb	0.51 ± 0.06 ^c^	0.95 ± 0.04 ^c^	1.86

Results are expressed as mean ± standard deviation; columns’ mean values with different letters are significantly different at *p* < 0.05.

**Table 2:** Total phenolic content (TPC), flavonoid content, and antioxidant capacity (ABTS) of the cryo-concentrated plant extracts used for determining the antiviral properties against MNV.

Plant Extract Source	TPC [mg GAE/100 mL]	Flavonoid [mg CE/100 mL]	ABTS [µmol Trolox/100 mL]	Virus-Inactivating Activity [%]
*Origanum vulgare* L. Oregano leaves	105.73 ^a^ ± 1.57	1217.94 ^a^ ± 2.12	574.56 ^c^ ± 4.34	99.90
*Coffea arabica* L. Arabica coffee roasted beans	77.67 ^b^ ± 3.14	955.28 ^a^ ± 0.56	451.15 ^d^ ± 5.21	99.91
*Cannabis sativa* L. Hemp seeds and leaves	47.26 ^c^ ± 17.81	268.58 ^d^ ± 2.76	791.31 ^b^ ± 5.21	99.95
*Thymus vulgaris* L. Common thyme leaves	37.69 ^c^ ± 1.32	455.28 ^b^ ± 1.14	762.45 ^b^ ± 0.87	99.99
*Urtica dioica* L. Common nettle leaves	25.53 ^d^ ± 2.15	335.03 ^c^ ± 4.7	818.32 ^a^ ± 3.47	99.99
*Rosmarinus officinalis* L. Rosemary leaves	24.83 ^d^ ± 3.14	357.18 ^c^ ± 7.01	807.27 ^a^ ± 5.21	99.99
*Zingiber officinale* Roscoe Ginger root	24.13 ^d^ ± 2.81	293.89 ^d^ ± 2.94	798.67 ^a^ ± 3.49	99.99
*Echinacea purpurea* L. Purple coneflower	14.54 ^d^ ± 0.5	300.22 ^d^ ± 3.01	832.44 ^a^ ± 4.34	99.99
*Cinnamomum verum* J. Presl Ceylon cinnamon bark	9.63 ^e^ ± 0.33	271.74 ^d^ ± 5.56	842.88 ^a^ ± 6.95	99.99
*Allium sativum* L. Garlic bulb	9.11 ^e^ ± 0.16	259.08 ^d^ ± 2.54	844.72 ^a^ ± 6.09	99.99

Results are expressed as mean ± standard deviation; mean values with different letters (a–e) are statistically different (*p* = 0.05).

**Table 3:** Concentrations of plant extracts used in antiviral studies determined based on cytotoxicity studies.

Substance Used for Testing	Concentration of the Test Substance (%)	Dilution that Did Not Produce Cytotoxic Effects
*Origanum vulgare* L. Oregano leaves	18	10^−3^
*Coffea arabica* L. Arabica coffee roasted beans	18	10^−3^
*Cannabis sativa* L. Hemp seeds and leaves	18	10^−3^
*Thymus vulgaris* L. Common thyme leaves	18	10^−3^
*Urtica dioica* L. Common nettle leaves	18	10^−3^
*Rosmarinus officinalis* L. Rosemary leaves	18	10^−3^
*Zingiber officinale* Roscoe Ginger root	18	10^−3^
*Echinacea purpurea* L. Purple coneflower	18	10^−3^
*Cinnamomum verum* J. Presl Ceylon cinnamon bark	18	10^−3^
*Allium sativum* L. Garlic bulb	18	10^−3^
Eicatechin (EPI)	10	10^−2^
Cavoylquinic acid (CQA)	10	10^−2^

**Table 4:** Parameters of extracts used for antiviral testing.

Substance Used for Testing	Extract Concentration % (*w*/*v*)	Concentration Used in Antiviral Tests (mg/mL)	The Volumes Used in EN 14476 Mixes	Solvent
*Origanum vulgare* L. Oregano leaves	2.04	9.7	800 μL	Dulbecco’s Modified Eagle’s Medium (DMEM)
*Coffea arabica* L. Arabica coffee roasted beans	2.04	9.9	800 μL	DMEM
*Cannabis sativa* L. Hemp seeds and leaves	2.04	10.1	800 μL	DMEM
*Thymus vulgaris* L. Common thyme leaves	2.04	9.3	800 μL	DMEM
*Urtica dioica* L. Common nettle leaves	2.04	7.7	800 μL	DMEM
*Rosmarinus officinalis* L. Rosemary leaves	2.04	9.4	800 μL	DMEM
*Zingiber officinale* Roscoe Ginger root	2.04	8.1	800 μL	DMEM
*Echinacea purpurea* L. Purple coneflower	2.04	9.6	800 μL	DMEM
*Cinnamomum verum* J. Presl Ceylon cinnamon bark	2.04	9.4	800 μL	DMEM
*Allium sativum* L. Garlic bulb	2.04	9.5	800 μL	DMEM

## Data Availability

Data will be made available on request.
